# The Passing of the General Practitioner

**Published:** 1909-11-27

**Authors:** 


The Hospital

A Journal of

The Medical Sciences and Hospital Administration.

New Series. No. 144, Vol. VI. [No. 1216, Vol. XLVII.] SATURDAY, NOVEMBER 27, 1909.

### THE PASSING OF THE GENERAL PRACTITIONER.

Many things are changing in this England of ours, to the no small loss of the people, though they wot it not. It is forty-four years since we commenced life as an apprentice to a practitioner in large practice in a great urban community. That is nearly five decades ago, when the general practitioner was a power in the land, and his rule was beneficent and good for the nation. He then obtained his qualifications after about three years' experience and study, if study it could be called, whereas to-day the medical student devotes five to seven years to study before he touches practice at all. It is remarkable to note that in 1865 and for many subsequent years the general practitioner was the trusted friend and counsellor of every family who called him in. To-day the general practitioner, with some few and notable exceptions, is too frequently passed over in favour of the consultant, and so he finds it harder and harder to make a living. This passing of the general practitioner, who was a tower of strength for the individual patient, should be resisted by every head of a household of prudence and intelligence. Paget wisely insisted on the dangers and responsibility of a disregard of conservatism in surgery. Wherever the general practitioner of the best type has proper authority, conservatism in surgery has just weight.

It may be useful to consider for a moment what factors have combined to lessen the confidence and authority formerly vested in the general practitioner. No doubt the facilities for travel, which make distance a matter of relative unimportance, has created a fashion whereby people consider it the thing to take a long journey and pay a fee to a consultant even in slight cases of illness. Another cause, we are confident, is the lowering of the profession in public esteem by the unwisdom of certain medical organisations which give the impression that the profession is becoming less of a profession and more of a trade or business every day. The degradation of practice through the mistaken efforts to live on the part of men who take smaller fees than the chemists for very indifferent treatment is another cause which has done much mischief. The abolition of apprenticeship has had a much greater influence for evil, so far as the status of the general practitioner is concerned, than is generally realised. The apprentice was caught young; he was brought directly into contact with the classes of people who must one day become his own patients; he learnt their habits and ways under the guidance of a master of experience and distinction, and he had the immeasureable advantage of acquiring much practical knowledge which the modern student rarely obtains. This disadvantage is aggravated by the circumstance that the present medical curriculum absorbs so much of the energies of students in mere study that practical work, a knowledge of the world and the handling of men and women, are too little regarded. This cramming of students' heads with knowledge obtained from books has changed the student's outlook on life, and left him when he receives his diploma pretty much where the University student is left on taking his degree. It has the further misfortune of making him more ambitious than his fellows to begin where his father left off, and so we have to-day this extraordinary position, in regard to the London schools of medicine especially, that nearly all the appointments at provincial hospitals are being taken by men educated at a Scottish or provincial school.

The passing of the general practitioner into the limbo of forgotten things would be an irretrievable loss to the profession and the public at large. In the sick-room, in the family, in the everyday life of his patients, the general practitioner of the past has been a power for good at least equal to that of the pastor or minister, and his moral influence has been a national asset. There is no thoughtful member of the profession of the widest experience who can quietly contemplate the threatened extinction of the general practitioner without serious apprehension. The general practitioner, by his knowledge of the patient and his history, was led to an accurate study of the nature of each case, which he was able to pass on to the consultant, whose trusted colleague and representative he thus became, when a second opinion became desirable. It is not too much to say that the passing of the general practitioner will mean the practical extinction of the physician. As Paget insisted and all the best minds in the profession know, such a result as this must be resisted by every wise and prudent member of the profession. Its consequences represent very real dangers to everybody concerned. Neither science nor the profession of medicine, nor the people of this or any country, unless the teachings of experience are to be treated with equal contempt to that exhibited by some for accurate diagnosis before active treatment, can contemplate the passing of the general practitioner without the gravest misgiving. We hope and believe that a way may be found to stay the passing of the general practitioner and to restore him to his former position of authority as the patient's protector and confidant. We have impressed upon the members of the profession and the managers of hospitals the responsibility of silence, and this article will, we hope, afford evidence that we recognise that journalists too have the same responsibility, which we do not intend to shirk.

